# Laparoscopic Treatment of Perforated Peptic Ulcer: A Propensity Score-Matched Comparison of Interrupted Stitches Repair versus Knotless Barbed Suture

**DOI:** 10.3390/jcm13051242

**Published:** 2024-02-22

**Authors:** Gianluca Costa, Giovanni Maria Garbarino, Luca Lepre, Gianluca Liotta, Gianluca Mazzoni, Alice Gabrieli, Alessandro Costa, Mauro Podda, Gabriele Sganga, Pietro Fransvea

**Affiliations:** 1 Surgery Center, Colorectal Surgery Research Unit, Fondazione Policlinico Universitario Campus Bio-Medico, University Campus Bio-Medico of Rome, 00128 Rome, Italy; g.costa@policlinicocampus.it; 2 General Surgery Unit, Sant’Eugenio Hospital, ASL Roma 2, 00144 Rome, Italy; 3 General and Emergency Surgery Unit, Santo Spirito in Sassia Hospital, ASL Roma 1, 00193 Rome, Italy; luca.lepre@aslroma1.it; 4 General and Emergency Surgery Unit, Palestrina Hospital, ASL Roma 6, 00036 Palestrina, Italy; 5 General Surgery Unit, G.B. Grassi Hospital, ASL Roma 3, 00122 Rome, Italy; 6 Department of General Surgery, Cattinara University Hospital, Azienda Sanitaria Universitaria Giuliano Isontina, 34148 Trieste, Italy; alice.gabrieli93@gmail.com; 7 UniCamillus School of Medicine, Saint Camillus International University of Health and Medical Sciences, 00131 Rome, Italy; 8 Department of Surgical Science, University of Cagliari, 09124 Cagliari, Italy; mauro.podda@unica.it; 9 Emergency Surgery and Trauma, Fondazione Policlinico Universitario “A. Gemelli” IRCCS, Catholic University of Sacred Heart, 00135 Rome, Italypietro.fransvea@policlinicogemelli.it (P.F.)

**Keywords:** perforated peptic ulcer, surgical treatment, laparoscopic approach, interrupted stitches suture, knotless barbed suture

## Abstract

**Background:** Peptic ulcers result from imbalanced acid production, and in recent decades, proton pump inhibitors have proven effective in treating them. However, perforated peptic ulcers (PPU) continue to occur with a persistent high mortality rate when not managed properly. The advantages of the laparoscopic approach have been widely acknowledged. Nevertheless, concerning certain technical aspects of this method, such as the best gastrorrhaphy technique, the consensus remains elusive. Consequently, the choice tends to rely on individual surgical experiences. Our study aimed to compare interrupted stitches versus running barbed suture for laparoscopic PPU repair. **Methods:** We conducted a retrospective study utilizing propensity score matching analysis on patients who underwent laparoscopic PPU repair. Patients were categorised into two groups: Interrupted Stitches Suture (IStiS) and Knotless Suture (KnotS). We then compared the clinical and pathological characteristics of patients in both groups. **Results:** A total of 265 patients underwent laparoscopic PPU repair: 198 patients with interrupted stitches technique and 67 with barbed knotless suture. Following propensity score matching, each group (IStiS and KnotS) comprised 56 patients. The analysis revealed that operative time did not differ between groups: 87.9 ± 39.7 vs. 92.8 ± 42.6 min (*p* = 0.537). Postoperative morbidity (24.0% vs. 32.7%, *p* = 0.331) and Clavien–Dindo III (10.7% vs. 5.4%, *p* = 0.489) were more frequently observed in the KnotS group, without any significant difference. In contrast, we found a slightly higher mortality rate in the IStiS group (10.7% vs. 7.1%, *p* = 0.742). Concerning leaks, no differences emerged between groups (3.6% vs. 5.4%, *p* = 1.000). **Conclusions:** Laparoscopic PPU repair with knotless barbed sutures is a non-inferior alternative to interrupted stitches repair. Nevertheless, further research such as randomised trials, with a standardised treatment protocol according to ulcer size, are required to identify the best gastrorraphy technique.

## 1. Introduction

The worldwide incidence of peptic ulcer disease has notably declined to 0.03% from 0.19%, following the introduction of proton pump inhibitors (PPI) alongside antibiotic therapy targeting Helicobacter pylori [1,2,3,4,5]. Despite a reduction in the number of ulcer patients to less than one-third of the past figure, there has not been a significant decrease in ulcer-related mortality. Perforated peptic ulcer (PPU) persists as a critical surgical emergency, contributing to about 35–40% of all deaths linked to peptic ulcers [6,7]. This suggests a shift towards more severe clinical presentations, notably exacerbated by combined use of low-dose aspirin (LDA), non-steroidal anti-inflammatory drugs (NSAIDs), and advanced age, all of which elevate the risk of LDA-induced ulcers, haemorrhage, and worsening conditions [8,9,10,11].

Surgical intervention remains the established treatment for PPU, either a primary repair or a free omental patch repair [12,13]. Since the first report of laparoscopic treatment of PPU in 1990, laparoscopy proved beneficial in terms of reduced intraoperative blood loss, improved pulmonary function, decreased postoperative pain, faster return of bowel function, shorter hospital stays, and a lower incidence of incisional hernias compared to conventional open surgery [14,15,16,17]. 

The progression of minimally invasive methods owes much to the ongoing dedication of surgeons, yet the significance of enhancements in surgical tools cannot be understated. A contributing element to the better results seen in laparoscopic surgery involves the knotless barbed suture. This innovation allows for the avoidance of laparoscopic suture knotting while reducing operative time of gastrointestinal surgery including PPU repair [18,19,20].

Our study aims to compare two different gastrorrhaphy techniques for laparoscopic PPU repair: interrupted stitches versus knotless barbed suture.

## 2. Material and Methods

### 2.1. Study Settings and Protocol

This study derives from previous research [17,21]. The IGo-GIPS (Italian Group for Gastro-Intestinal Postoperative Surveillance) is a nationwide network created with the aim to investigate the perioperative outcomes of specific topics mainly concerning emergency gastrointestinal surgery [22]. Clinical decisions, including operative technique, were always based on the criteria of individual centres and staff surgeons. Although procedures were not standardised per a study protocol, it is important to note that they were likely similar among participating hospitals, with some slight technical differences across institutions seldom taken into account because they were judged to not influence the outcome. All the investigators were informed about the objectives of the project and asked for complete details about the management of patients. The protocols were already extensively described [21,22]. Data regarding patients were prospectively collected from the study participating centres from January 2017 to June 2018, while data regarding patients from July 2018 to June 2023 were retrieved either retrospectively from hospital electronic databases or prospectively collected following well-designed studies. Both the prospective study protocols [21,22] were approved by the Ethics Committees, the former by Sapienza University of Rome and the latter by University Campus Bio-Medico of Rome. No formal approval was requested for any other retrospective non-interventional study except in case of specific indication deemed by a single centre. However, a signed consent for the storage and analysis of personal data for scientific purposes was obtained from all patients upon hospital admission. This study was conducted in accordance with the Declaration of Helsinki and its later amendments. All parts of the studies and the present manuscript have been checked and presented according to the checklist for Strengthening the Reporting of Observational Studies in Epidemiology (STROBE) [23]. 

### 2.2. Inclusion and Exclusion Criteria and Collected Data Confirmation

For the aim of the present study, we initially retrieved records of all patients >18 years with ICD-9-CM code ranging from 531.x to 534.x requiring emergency surgery from January 2017 to June 2023. Furthermore, bleeding ulcer, neoplastic perforation, sole endoscopic procedures, and emergency operations during the course of any other elective surgery were discarded. Other exclusion criteria were the following: upfront open surgery; laparoscopic procedure converted to open surgery; location other than prepyloric region or duodenal bulb; use of topical adhesives and/or surgical sealants as healing adjunct; previous upper GI surgery; lack of informed consent, if requested; and patients participating in other randomised or interventional clinical trials. Moreover, submissions made by unconfirmed participants, duplicate submissions, unspecified, unclear or mixed technique performed, and records with more than 5% of missing data were also excluded. Finally, only patients submitted to one-layer repair with interrupted stitches or knotless suture by laparoscopic approach were considered. No exclusion criteria were adopted regarding the use of the omentum. Due to the wide variability defining repair procedures found in the literature, the surgical techniques were grouped as follows: simple suture, suture plus any modified omental patch, and Graham omentopexy, as described by Demetriou and Chapman [24]. Although the patients’ demographic information was collected, raw data were managed and anonymised before analysis even for centre identification by an IT specialist not involved in the research. 

### 2.3. Patients’ Characteristics, Preoperative Variables and Objectives of This Study

Patients were divided into two groups named Interrupted Stitches Suture (IStiS) and Knotless Suture (KnotS). Clinical-pathological features of patients in both groups were compared. Data collected included patient demographic characteristics and clinical variables, procedure details, and outcomes. Demographics variables and clinical data included: age, gender, weight, height, body mass index (BMI), Glasgow Coma Scale (GCS), heart rate, systolic blood pressure, medical and surgical history (comorbidities), and common preoperative biochemical blood examination (including C-Reactive Protein [CPR], and arterial blood gas analysis). Procedure details included: site and size of the ulcer, timing, and peritoneal contamination. Comorbidity was recorded if the condition was being medically treated at the time of admission, or if previous treatment for the condition was described in the admission report. The Age-adjusted Charlson Comorbidity Index (age-CACI) was calculated, and a score ≥ 6 was used to categorise patients with a severe comorbid condition. Preoperative risk was assessed with anaesthesiologist-assigned American Society of Anaesthesiologists (ASA). Furthermore, systemic inflammatory response syndrome (SIRS) was evaluated according to the original consensus study (Sepsis-1) [25]. SIRS criteria ≥ 2 met the definition of SIRS. When appropriated, the frailty profile was investigated either by the 5-modified Frailty Index (5-mFI) or by the Emergency Surgery Frailty Index (EmSFI), as already described [26]. When statistical analysis was performed, 5-mFI ≥ 0.4 score was used for categorising frailty as a binary variable according to the current literature [27]. Sepsis was evaluated according to the qSOFA score. The Shock Index, the Age–Shock Index, the Mannheim Peritonitis Index (MPI), the Boey score, and the PULP Score were also calculated. Postoperative complications have been reported and categorised according to the Clavien–Dindo classification system by the study leader in each of the participating centres, and the Comprehensive Complication Index was calculated. This variable was evaluated in two different ways: (a) as a continuous variable in all patients from 0 to 100 scale and (b) as a continuous variable only if CCI was ≥8 (at least one C-D I) [28,29]. Furthermore, morbidity was divided into three groups as follows: C-D I-II, C-D III, and C-D IV. Clavien–Dindo Grades III and IV were also defined as major complications. Although morbidity and mortality have been considered as the 30-day standard period definition, adverse outcomes have been reported regardless of the time elapsed from the surgical procedure if reasonably related to it and occurred during hospitalisation following the main emergency procedure. Leakage was defined as when bile or gastric content was detected in the drain output, at CT scan with oral water-soluble contrast, or during reoperation. No routine use of CT scan or of the methylene blue test was adopted. As well as stated above, due to the design of this research, there was not a uniform standardised protocol neither for the whole technique performed nor for the suture material used. However, because the most common equally distributed procedure performed in both groups was the suture plus omental patch, the lack of uniformity in the technique and material used was not considered as a bias. 

## 3. Statistical Analysis

Statistical analysis was carried out using StataCorp 2019 STATA Statistical Software: release 16 (StataCorp LLC, College Station, TX, USA). Initially, the findings of all patients in the two groups were evaluated. Dichotomous data and counts were presented in frequencies, whereas continuous data were presented as mean values ± standard deviations (SD) and/or median with 25–75 Interquartile Range (IQR) and minimum–maximum range. Differences between means were compared using the independent sample Student’s *t*-test or the Mann–Whitney U test when indicated. Fisher’s exact test or χ^2^ test, with or without Yates correction, were used to compare differences in frequencies.

Thereafter, a propensity score matching was carried out. The Italian Version of IBM Corp. Released 2012 SPSS Statistics for Macintosh, Version 21.0. IBM Analytics (Segrate, Milan, Italy) integrated with SPSS R Essentials for R Statistical Software version 2.14.2 (Foundation for Statistical Computing, Vienna, Austria) was used. The model was constructed to eliminate selection bias between groups as recommended [30]. Variables with potential influence on outcomes were assigned propensity scores using a bivariate logistic regression model. The final model included the following variables: sex as exact, age, BMI, Creatinine, Age-CACI, EmSFI, and 5-mFI. We matched propensity scores 1:1 with the use of the nearest neighbour methods without replacement using the closest calliper width to achieve the maximum number of cases without statistical differences in confounders. In this instance, the calliper width was set at 0.2. All tests were two-tailed, and a *p* value ≤ 0.05 was considered statistically significant. 

## 4. Results

### 4.1. Entire Series

**Figure 1:**
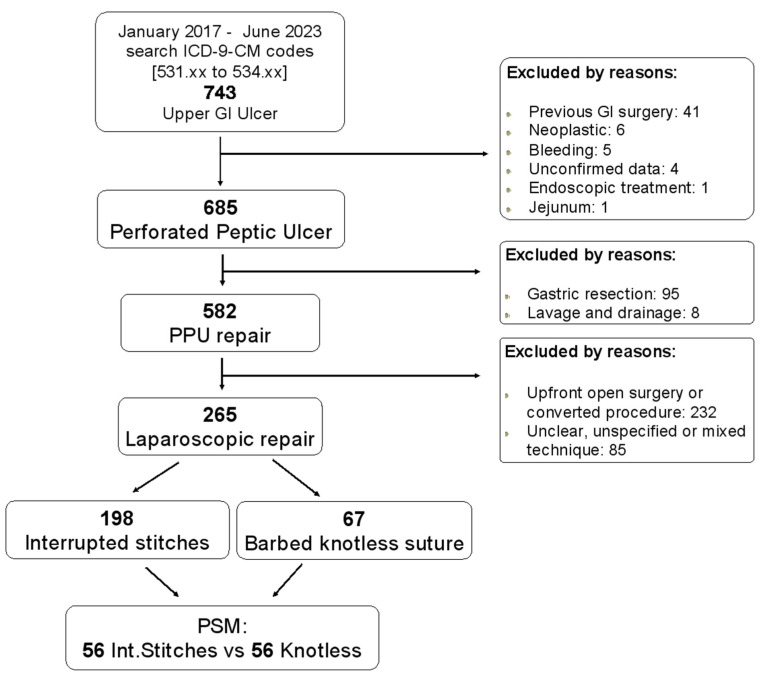
The PRISMA flowchart.

A total of 265 patients fulfilling the inclusion criteria were evaluated. A flow chart is represented in Figure 1. The overall mean age was 60.6 ± 16.8 (range, 18 to 92 years); as regard to sex, 144 (54.3%) patients were male. An interrupted stitches repair (IStiS) was performed in 198 patients (74.7%) while a barbed knotless suture (KnoS) was performed in 67 patients (25.3%). In total, 5 patients (1.9%) underwent simple repair, 256 (96.6%) were submitted to repair plus omental patch, and 4 patients (1.5%) underwent Graham omentopexy. The mean operative time was 91.7 ± 41.2 min. The overall morbidity and mortality rates were 28.9% and 9.8%, respectively. Leakage occurred in 17 patients (6.4%) with associated mortality of 35.3%. 

### 4.2. Comparison before PSM

The rate of male patients was similar between the groups as well as age, BMI, and ASA score. No differences were found between the two groups in terms of preoperative laboratory value (Hb, lactate, glycemia, WBC, PLT CRP) except for creatinine, which was significantly higher in the KnotS patients (KnotS 1.30 ± 1.04 vs. IStiS 1.01 ± 0.64; *p* < 0.007). 

Boey score, Pulp score, and MPI were not different between the groups. Regarding the scores that reflect patient general condition upon arrival, we found that the Shock Index, Age–Shock Index, SIRS, and qSOFA were similar. Nevertheless, patients in the KnotS group had more comorbidities (Age-CACI: KnotS 3.10 ± 2.37 vs. IStiS 2.46 ± 2.26; *p* = 0.048) and were more fragile, as reflected by significantly higher EmSFI and 5-mFI indices, and a higher rate of frailty (19.4% vs. 11.1% *p* = 0.083; OR 1.926; [95% Conf. Interval 0.904–4.102]). (Table 1 and Table 2)

The mean operative time was 92.0 ± 41.6 min in the IStiS group and 90.8 ± 40.1 min in the KnotS group. The difference was not statistically significant. The mean diameter of the perforation and site were similar between the groups, and no difference in terms of leak rate was retrieved. 

Operative details and postoperative outcomes before and after propensity score matching are summarised in Table 3. In the KnotS group, the overall morbidity rate was slightly higher, almost reaching statistical significance (IStiS 46 patients (25.7%) vs. KnotS 23 patients (38.3%) *p* = 0.062; OR 1.798; [95% Conf. Interval 0.962–3.357]). 

Major complications occurred in 15 patients (7.6%) in the IStiS group: 8 patients had C-D grade III complication. Five patients had IIIb type (four leaks treated in two cases by successful resuture and in two cases by gastric resection, and one pleural empyema underwent thoracoscopy), and three patients had IIIa complication (two subphrenic abscesses and one intra-abdominal bleeding due to splenic injury treated by angio-embolisation). Seven patients had C-D grade IV complication (three pneumonia with respiratory insufficiency, two acute renal failure requiring dialysis, one myocardial infarction, one ischemic stroke).

Major complications occurred in nine patients (13.4%) in the KnotS group: six patients had C-D grade IIIb complication (one leak treated by successful resuture, one leak underwent gastrectomy, and one small bowel obstruction), and three IIIa cases were observed (one upper GI bleeding treated by endoscopic haemostasis [Dieulafoy’s lesion], one subphrenic abscess, one iatrogenic pneumothorax). Three patients had C-D grade IV complication (one acute renal failure requiring dialysis, one myocardial infarction, and one DVT with pulmonary embolism and pneumonia). 

Major complications were more frequently observed in the KnotS group, but the difference was not statistically significant (13.4% vs. 7.6%, *p* = 0.231; OR 1.893; [95% Conf. Interval 0.783–4.576]). The overall postoperative 30-day mortality rates were similar between groups with a slightly higher rate in the KnotS group. 

### 4.3. Comparison after PSM

After the propensity score matching (PSM) procedure, 56 patients of the IStiS group and 56 patients of the KnotS group were selected for comparison. The analysis revealed that the pre-operative variables found to be significantly different before matching (i.e., Creatinine, CACI, and EmSFI) were then well balanced (Table 2). Similarly, no difference was noted between the groups regarding comorbidities, frailty, and operative details. Again, there was no difference in terms of leak rate between the groups, and there were no statistically significant differences in morbidity and mortality. However, following PSM, we found a slightly higher mortality rate in the IStiS group as opposed to what was observed before propensity. While focusing on major complications, we noted that there were no C-D IV complications in either group. Although Clavien–Dindo III were more frequently observed in the KnotS group, the rates were comparable between the two groups and the difference was not significant. (10.7% vs. 5.4%, *p* = 0.489; OR 2.120; [95% Conf. Interval] 0.496–9.058). In the IStiS group, we retrieved one leak treated by successful resuture, and two subphrenic abscesses, while in the KnotS group, six patients had C-D grade III complications as reported above. Demographic characteristics, procedure details, and post-operative course of patients pre and post propensity matching study are shown in Table 1, Table 2 and Table 3.

**Table 1:** General demographics characteristics and clinical data (IStiS: interrupted stitches; KnotS: knotless barbed suture).

	Entire Cohort 265 (%)	IStiS 198 (%)	KnotS 67 (%)	*p* Value
**Gender, male n. (%)**	144 (54.3)	109 (55.1)	35 (52.2)	0.690
**Mean age, (range)**	60.6 ± 16.8 (18–92)	59.9 ± 17.1 (18–92)	62.4 ± 16.0 (29–91)	0.291
**BMI**	25.2 ± 4.6	25.4 ± 4.7	24.7 ± 4.5	0.342
**ASA ≥ 3**	123 (46.4)	92 (46.5)	31 (46.3)	0.978
**Site**				0.848
*Gastric prepyloric*	116 (43.8)	86 (43.4)	30 (44.8)	
*Duodenal bulb*	149 (56.2)	112 (56.6)	37 (55.2)	
**Size (mm)**	7.7 ± 2.6	7.8 ± 2.6	7.7 ± 2.6	0.750
**Surgical procedure**				
*Simple suture*	17 (6.4)	12 (6.1)	5 (7.5)	
*Suture plus omental patch*	239 (90.2)	179 (90.4)	60 (89.5)	
*Graham omentopexy*	9 (3.4)	7 (3.5)	2 (3.0)	
**Operating time (minutes)**	91.7 ± 41.2	92.0 ± 41.6	90.8 ± 40.1	0.832
**Hemoglobin**	13.7 ± 2.5	13.7 ± 2.3	13.5 ± 3.0	0.604
**WBC**	13.5 ± 5.6	13.3 ± 5.4	13.9 ± 6.0	0.417
**PLT**	279.7 ± 89.2	281.4 ± 91.4	274.7 ± 83.0	0.598
**Glycemia**	139.9 ± 46.0	140.0 ± 49.1	139.6 ± 35.9	0.948
**Creatinine**	1.1 ± 0.8	1.0 ± 0.6	1.3 ± 1.1	**0.007**
**INR**	1.2 ± 0.5	1.2 ± 0.5	1.2 ± 0.6	0.538
**Lactate (mmol/L)**	2.8 ± 0.1	2.7 ± 0.1	2.9 ± 0.2	0.403
**C-Reactive Protein**	6.5 ± 6.9	6.6 ± 7.2	6.2 ± 6.0	0.707
**BOEY score**	1.2 ± 0.9	1.2 ± 1.0	1.3 ± 1.0	0.383
**Mannheim Peritonitis Index (MPI)**	17.1 ± 7.8	17.2 ± 8.1	17.0 ± 6.7	0.866
**Pulp Score**	4.3 ± 3.0	4.2 ± 3.0	4.4 ± 3.1	0.717
**SIRS**	1.3 ± 1.0	1.3 ± 0.9	1.4 ± 1.0	0.418
**qSOFA**	0.29 ± 0.03	0.28 ± 0.03	0.31 ± 0.08	0.685
**Shock Index**	0.7 ± 0.2	0.7 ± 0.2	0.7 ± 0.2	0.958
**Age–Shock Index**	42.3 ± 19.1	41.7 ± 18.4	44.2 ± 21.2	0.354
**CACI**	2.62 ± 2.29	2.46 ± 2.25	3.10 ± 2.36	**0.049**
**CACI ≥ 6**	32 (12.1)	21 (10.6)	11 (16.4)	0.207
**EmSFI**	2.86 ± 1.29	2.75 ± 1.18	3.17 ± 1.53	**0.021**
**5-Item frailty Index**	0.12 ± 0.16	0.11 ± 0.14	0.17 ± 0.19	**0.004**
**Frailty yes (5-mFI ≥ 0.4)**	35 (13.2)	22 (11.1)	13 (19.4)	0.083
**LOS (days)**	10.5 ± 10.7	10.3 ± 10.8	11.3 ± 10.4	0.518
**Morbidity**	69 (28.9)	46 (25.7)	23 (38.3)	0.062
*Clavien–Dindo I–II*	46 (17.4)	32 (16.2)	14 (20.9)	0.377
*Clavien–Dindo III*	14 (5.3)	8 (4.0)	6 (9.0)	0.126
*Clavien–Dindo IV*	10 (3.8)	7 (3.5)	3 (4.5)	0.717
**CCI (Comprehensive Complication Index 0–100)**	47.35 ± 34.59	48.97 ± 35.12	43.84 ± 33.74	0.504
**CCI (Comprehensive Complication Index ≥8)**	17.05 ± 30.70	16.18 ± 30.51	19.63 ± 31.35	0.428
**Mortality**	26 (9.8)	19 (9.6)	7 (10.5)	0.815

Significant results (*p* > 0.05) were marked with bold.

**Table 2:** Demographics characteristics and clinical data of IStiS (interrupted stitches) and KnotS (knotless barbed suture) groups before and after propensity score matching.

	Before Propensity Score Matching		*p* Value	After Propensity Score Matching		*p* Value
	IStiS 198 (%)	KnotS 67 (%)		IStiS 56 (%)	KnotS 56 (%)	
**Age, year**	59.9 ± 17.1	62.4 ± 16.0	0.291	63.6 ± 15.1	62.5 ± 15.3	0.715
**Male sex n (%)**	109 (55.1)	35 (52.2)	0.690	30 (53.6)	30 (53.6)	1.000
**BMI, kg/m^2^**	25.4 ± 4.7	24.7 ± 4.5	0.342	25.2 ± 5.8	24.4 ± 4.7	0.441
**ASA ≥ 3**	92 (46.5)	31 (46.3)	0.978	23 (41.1)	24 (42.9)	0.251
**Lactate (mmol/L)**	2.7 ± 0.1	2.9 ± 0.2	0.403	2.5 ± 0.2	2.8 ± 0.2	0.374
**Glycemia**	140.0 ± 49.1	139.6 ± 35.9	0.948	148.8 ± 62.1	141.8 ± 35.5	0.470
**Creatinine**	1.0 ± 0.6	1.3 ± 1.1	**0.007**	1.0 ± 0.4	1.1 ± 0.8	0.301
**INR**	1.2 ± 0.5	1.2 ± 0.6	0.538	1.3 ± 0.5	1.2 ± 0.6	0.899
**Hemoglobin (g/dL)**	13.7 ± 2.3	13.5 ± 3.0	0.604	13.5 ± 2.4	13.9 ± 2.3	0.304
**WBC (10^9^/L)**	13.3 ± 5.4	13.9 ± 6.0	0.417	13.4 ± 5.2	13.6 ± 6.2	0.828
**PLT**	281.4 ± 91.4	274.7 ± 83.0	0.598	289.9 ± 108.8	274.9 ± 83.5	0.413
**C-Reactive Protein (mg/L)**	6.6 ± 7.2	6.2 ± 6.0	0.707	6.1 ± 6.8	6.1 ± 6.2	0.976
**CACI**	2.46 ± 2.25	3.10 ± 2.36	**0.049**	2.78 ± 1.96	2.73 ± 2.09	0.889
**CACI ≥ 6**	21 (10.6)	11 (16.4)	0.207	4 (7.1)	5 (8.9)	1.000
**Shock Index**	0.7 ± 0.2	0.7 ± 0.2	0.958	0.7 ± 0.2	0.7 ± 0.2	0.676
**Age-Shock Index**	41.7 ± 18.4	44.2 ± 21.2	0.354	45.0 ± 17.6	43.3 ± 17.9	0.611
**SIRS**	1.3 ± 0.9	1.4 ± 1.0	0.418	1.4 ± 1.0	1.3 ± 1.0	0.852
**qSofa**	0.28 ± 0.03	0.31 ± 0.08	0.685	0.30 ± 0.07	0.26 ± 0.08	0.745
**EmSFI**	2.75 ± 1.18	3.17 ± 1.53	**0.021**	2.82 ± 0.79	2.89 ± 1.02	0.679
**5-Item frailty Index**	0.11 ± 0.14	0.17 ± 0.19	**0.004**	0.12 ± 0.13	0.14 ± 0.16	0.601
**Frailty yes (5-mFI ≥ 0.4)**	22 (11.1)	13 (19.4)	0.083	3 (5.4)	7 (12.5)	0.321

Significant results (*p* > 0.05) were marked with bold.

**Table 3:** Operative details and postoperative outcomes of IStiS (interrupted stitches) and KnotS (knotless barbed suture) groups before and after propensity score matching.

	Before Propensity Score Matching		*p* Value	After Propensity Score Matching		*p* Value
	IStiS 198 (%)	KnotS 67 (%)		IStiS 56 (%)	KnotS 56 (%)	
**Operative time (min), mean ± SD**	92.0 ± 41.6	90.8 ± 40.1	0.832	87.9 ± 39.7	92.8 ± 42.6	0.537
**Site**			0.848			0.131
*Gastric prepyloric*	86 (43.4)	30 (44.8)		32 (57.1)	24 (42.9)	
*Duodenal bulb*	112 (56.6)	37 (55.2)		24 (42.9)	32 (57.1)	
**Ulcer size (mm), mean ± SD**	7.8 ± 2.6	7.7 ± 2.6	0.750	7.6 ± 2.4	7.8 ± 2.7	0.756
**Boey Score**	1.2 ± 1.0	1.3 ± 1.0	0.383	1.2 ± 1.0	1.2 ± 0.9	0.774
**Mannheim Peritonitis Index (MPI)**	17.2 ± 8.1	17.0 ± 6.7	0.866	17.1 ± 7.3	16.8 ± 6.9	0.842
**Pulp score**	4.2 ± 3.0	4.4 ± 3.1	0.717	4.5 ± 2.7	4.2 ± 2.9	0.569
**Leak**	12 (6.1)	5 (7.5)	0.773	2 (3.6)	3 (5.4)	1.000
**30 days morbidity (Clavien–Dindo I–IV) (n, %)**	46 (25.7)	23 (38.3)	0.062	12 (24.0)	17 (32.7)	0.331
*Clavien–Dindo I–II*	32 (16.2)	14 (20.9)	0.377	10 (17.9)	11 (19.6)	0.809
*Clavien–Dindo III*	8 (4.0)	6 (9.0)	0.126	3 (5.4)	6 (10.7)	0.489
*Clavien–Dindo IV*	7 (3.5)	3 (4.5)	0.717	-	-	
**CCI (Comprehensive Complication Index 0–100)**	48.97 ± 35.12	43.84 ± 33.74	0.504	48.88 ± 38.35	38.68 ± 32.68	0.375
**CCI (Comprehensive Complication Index ≥8)**	16.18 ± 30.51	19.63 ± 31.35	0.428	16.09 ± 31.32	14.50 ± 27.30	0.776
**Length Hospital Stay (days, median)**	10.3 ± 10.8	11.3 ± 10.4	0.518	9.4 ± 8.1	10.8 ± 10.6	0.465
**Postoperative 30-day mortality, n (%)**	19 (9.6)	7 (10.5)	0.815	6 (10.7)	4 (7.1)	0.742

## 5. Discussion

The advancement in laparoscopic surgical expertise alongside the development of various laparoscopic tools and modern anaesthesia techniques has significantly improved the safety and feasibility of laparoscopic procedures. Consequently, there has been a widespread adoption of minimally invasive approaches, even for complex and challenging operations, particularly in emergency settings [31,32,33].

PPU presents an ideal scenario for laparoscopic intervention due to its straightforwardness. The laparoscopic approach allows for easy identification of the perforation site, primary or omental patch repair and peritoneal lavage.

Although laparoscopic repair of PPU was documented as early as 1990, the adoption rates for this approach have been variable [34]. International studies report laparoscopic repair rates ranging from 41% to 76%, a range consistent with our previous findings [17,21,35,36]. Additionally, the 2020 WSES guidelines suggest the laparoscopic approach as the primary treatment for stable patients with small ulcers, provided surgeons possess the necessary skills and appropriate equipment [37]. 

The surgical strategy for perforated peptic ulcers has undergone substantial changes over the years. In the 1960s, the predominant procedure ranged from vagotomy and pyloroplasty to partial gastrectomy, associated with inherent risks [38,39,40]. However, in the subsequent decades, a less aggressive approach recommending simple suture with or without omentoplasty, omental pedicle flap (Cellan-Jones repair), free omental plug (Graham patch), or jejunal serosa patch gained traction together with a laparoscopic approach [41,42,43,44,45,46,47,48]. Modifications in surgical techniques, such as the use of fibrin glue, automated stapler devices, and continuous suture closure, have evolved to streamline the procedure and reduce operative complexity. There exists considerable variation in practices among surgeons and institutions regarding these approaches. 

The successful implementation of laparoscopic PPU repair is also owed to the development of new suture materials, notably knotless barbed sutures [20]. Despite some drawbacks like higher costs and irreversibility, knotless barbed sutures have gained acceptance in general surgery [49,50].

Clinical experiences report reduced procedure times and comparable complication rates in laparoscopic bowel suturing using these sutures [51,52]. Their evenly spaced barbs along the strand distribute tension evenly, promoting good blood supply at the sutured site. The absence of a knot and the design of the welded loop anchor simplify laparoscopic suturing, saving time and effort. 

Despite numerous studies validating the safety and efficacy of laparoscopic approaches for treating PPU, the consensus remains elusive regarding the best gastrorraphy technique [35,37]. 

In the ongoing debate, this manuscript showcases the non-inferiority of knotless barbed suture compared to interrupted stitches for laparoscopic PPU repair through a propensity score matching analysis. This approach, alongside randomisation, stands as the most robust method available for mitigating selection bias when evaluating outcomes associated with surgical techniques. Thus, this study aims to provide high-quality and dependable evidence in support of both gastrorraphy techniques. 

Among the interesting results of our study, we noticed a more fragile population in the KnotS group before matching. We hypothesised that surgeons were influenced by the frailty status of the patient when choosing the gastrorraphy technique. The surgeons probably opted for continuous suturing in the more fragile patients thinking that this technique could shorten the operative time.

This hypothesis was later discredited both by the results of propensity score matching, which showed no difference in frailty between the two groups, and by the results regarding operative time.

In contrast with previous research, our study showed comparable operative time using knotless barbed sutures compared to conventional interrupted stitches [18,19,20]. One possible explanation could be the laparoscopic skill of the surgeons. In fact, all participating surgeons had already completed their learning curve for laparoscopic gastrointestinal surgery at the beginning of the study. Moreover, considering the average size of the ulcer, usually two or three interrupted stitches were sufficient for an adequate gastrorrhaphy. 

Regarding morbidity, the literature reports approximately 30% incidence of postoperative complications. Our morbidity rate aligns with this statistic, with a slightly better morbidity rate for the IStiS group. Curiously, the mortality rate was not significantly lower in the KnotS group. Suture leak is the most feared complication and the major cause of reoperation after surgical repair. Proposed explanations from the current literature include difficulties in laparoscopic knot tying, ulcer diameter (>2 cm), and abdominal contamination [53,54]. Our previous multivariate analysis identified the ulcer site (pyloric/duodenal), a higher Boey Score, and a higher Age–Shock Index as factors associated with leaks [21]. Concerning leaks, the present analysis did not show any difference between the two gastrorrhaphy techniques. Chou et al. recently recorded a slightly increased leakage rate in the barbed suture group and hypothesised that the key point of the problem was leakage at the corner of the suture [20]. Effectively barbed sutures often need a couple of bites to fix the wire before addressing the defect to close. Accordingly, they modified their procedure, focusing on starting the suture at the perforation’s apex as much as possible. Consequently, these adjustments led to a significant decrease in the complications associated with leaks [20].

## 6. Limitations and Conclusions

The current study has several limitations. First, it encompasses both prospective and retrospective data collected from multiple centres, and therefore lacks a pre-established standardised treatment protocol.

Despite efforts to maintain uniform data collection, variations in the timing and choice of the gastrorraphy technique might have arisen due to differences in attending surgeons’ preferences, expertise, and intra- or inter-hospital settings. Consequently, the analysis could not be stratified by participating surgeons or institutions due to ethical considerations in the study protocol Additionally, challenges persist regarding the size of perforation and surrounding tissue quality, influencing surgeons’ decisions during repair. 

Moreover, while a multicentre study allows for broader generalisation of results compared to a single-centre study, the use of a propensity score model enables the comparison of similar restricted groups, minimizing confounding variables and addressing undetectable selection biases.

Lastly, this study did not delve into long-term outcomes, such as the recurrence of perforated peptic ulcers, or bleeding, which could offer valuable insights into the sustained efficacy of the chosen gastrorraphy technique.

In conclusion, continual advancements in surgical techniques are essential for both surgeons and patients. Based on the results of the present study, we can support that laparoscopic running barbed knotless suture of perforated peptic ulcers is a safe technique showing its non-inferiority when compared with the interrupted stitches technique. 

Nevertheless, further research such as randomised trials, with a standardised treatment protocol according to the ulcer size, are required to identify the best gastrorraphy technique.

## Data Availability

Data are contained within the article

## Appendix A. List of Italian Group for Gastro-Intestinal Surgery Postoperative Surveillance (IGo-GIPS)

Agresta F, Alemanno G, Antropoli M, Apice N, Argenio G, Avenia N, Azzinnaro A, Barberis A, Badessi G, Baldazzi G, Bergamini C, Bianco G, Biloslavo A, Bombardini C, Borzellino G, Brachini G, Buonanno GM, Canini T, Capolupo GT, Carannante F, Caricato M, Cassini D, Castriconi M, Catamerò A, Catarci M, Ceccarelli G, Ceresoli M, Chiarugi M, Cillara N, Cirocchi R, Cobuccio L, Coccolini F, Cocorullo G, Colutta C, Costa A, Costa G, Cozza V, Crucitti A, Cucinotta E, D’Alessio R, de Manzoni Garberini A, De Nisco C, De Prizio M, Finotti E, Floris F, Fransvea P, Frezza B, Gabrielli A, Garbarino GM, Garulli G, Genna M, Giannessi S, Giordano A, Grieco M, Grondona P, Guerrieri M, Iacopini V, Iarussi T, Kurihara H, Lagreca A, Laracca GG, Laterza E, Lepre L, Liotta G, Mariani D, Marini P, Marzaioli R, Mascianà G, Mercantini P, Milacci V, Mingoli A, Miranda G, Occhionorelli S, Paderno N, Palini GM, Paradies D, Petruzzelli L, Pezzolla A, Piazza D, Piazza V, Pignata G, Pinotti E, Pisanu A, Podda M, Prosperi P, Puccioni C, Rocca A, Romagnoli A, Romairone E, Rondelli F, Ruscelli P, Sandano M, Sapienza P, Scatizzi M, Serao A, Sganga G, Tartaglia D, Tebala G, Tranà C, Zago M.
